# Editorial: Sex Difference in Cancer Genomics and Its Impact on Therapy

**DOI:** 10.3389/fgene.2021.815804

**Published:** 2021-12-16

**Authors:** Sen Peng, Kristin Waite, Joshua B. Rubin, Jill S. Barnholtz-Sloan

**Affiliations:** ^1^ Cancer and Cell Biology Division, The Translational Genomics Research Institute, Phoenix, AZ, United States; ^2^ Trans-Divisional Research Program, Division of Cancer Epidemiology and Genetics, NCI, Shady Grove, MD, United States; ^3^ Departments of Pediatrics and Neuroscience, Washington University School of Medicine, Saint Louis, MO, United States; ^4^ Center for Biomedical Informatics and Information Technology, NCI, Shady Grove, MD, United States

**Keywords:** sex diferences, cancer genomcis, therapy, epidemiology, mechanisms

Most cancers occur more frequently in males compared to females. Moreover, for many cancers, females survive longer after their cancer diagnosis when compared to males. While the biological underpinnings of sex differences in cancer incidence and survival are not well understood, they must be traceable to the genetic effects of differing sex chromosome complement, *in utero* sex hormone epigenetic actions, and the activational effects of circulating sex hormones post-puberty. Together these constitute the mechanisms of sexual differentiation which affects all scales of biology, from cellular to body systems, levels.

In this Sex Difference in Cancer Research Topics, we received review articles as well as a variety of original research articles that comprehensively investigated clinical, epidemiological and genomic details of sex difference in cancer both in general as well as in specific tumor types.

In their review, Lopes-Ramos et al. summarized the genetic and genome-wide influences of sex in cancer and the impact of these influences on the trajectory of disease. Specifically, they reviewed how the following contribute to sex differences in cancer: genetics (SNP & somatic mutations, where there are increased cancer risk for females with TP53 mutations and higher mutational burdens in male cancers), epigenetics (sex-biased methylation patterns in 13 cancer types and sex-biased chromatin accessibility), gene regulation and expression (sex difference in eQTLs potentially due to allelic direction or gene expression variance but not the mean expression itself and regulatory disparity in miRNA & gene networks). The authors highlight the importance of integrative analyses in understanding the interplay of sex and genomics in cancer.

Haupt and Haupt performed a comprehensive review of the role of p53 and how sex modulates its critical role in cancer sex disparities. Their review examines the question of why the somatic *TP53* mutation incidence is estimated to be disproportionately higher among males then females. Further, the authors set forth what is known with regards to sex difference mutations & SNPs inTP53 as well as its regulators, the role of sex differences in methylation, post-translational modifications due to kinase modifications and glycosylation, the roles of non-coding RNAs (lncRNA and X-Chromosome MicroRNAs) and finally, the role of redox activity through the p53-ROS axis and cellular energy production. This comprehensive review raises the importance of the breadth of factors that may influence p53 function disparately in males and females.

For additional follow-up studies, the editors would recommend a) utilizing additional datasets that would allow for integrative analyses for investigation of sex differences in cancer and b) additional interrogation of datasets that would allow further delineation of the breadth of factors that may influence sex differences in p53 function.

Using a meta-analysis approach, Li et al. investigated the epidemiological characteristics of lung adenocarcinoma presenting with ground glass opacity (GGO). The recognition of this patient subgroup has increased in parallel with the availability of low dose computed tomography. They found that females comprise the majority of these patients, who are also distinguished from other lung adenocarcinoma patients by their lower age (∼59 vs. ∼66 years), and increased proportion of non-smokers. The authors’ analysis suggests that risk assessment of current lung cancer screening criteria may warrant a reassessment.

In their original research paper, Xue et al. preformed a retrospective analysis of gene mutations in Chinese patients with lung cancer. In general, the majority (51.8%) of lung cancer patients were greater than 60 years of age with a higher occurrence in females; 55.38% of cases occurred in females compared to 44.62% of cases in males. They found that overall, mutations were identified in EGFR, KRAS, ALK, BRAF, HER2, and PIK3CA and identified several sex specific differences in their analysis. When analyzing sex-differences in the mutation status in Chinese lung cancer patients they found that females predominately had mutations in EGFR while males predominately had mutations in KRAS. Sex-differences were also observed when stratifying for smoking history. Male lung cancer patients were predominately smokers (49.4%), in contrast to female lung cancer patients where 78.9% were non-smokers.

Liang et al., sought to identify novel single nucleotide polymorphisms (SNPS) in PDCD1 and its ligands CD274 and PDCD1LG2. They performed GWAS analysis in a multi-center case-control study comprised of never-smoking females and healthy women in Taiwan. Analysis of clinical data demonstrated that lung adenocarcinoma in never-smoking females was associated with: low education levels, history of lung cancer in a first-degree family member, previous tuberculosis infection, amount of cooking and cooking fume exposure, and environmental exposure to smoke. The authors identified twelve PDCD1LG2 SNPs there were associated with lung adenocarcinoma in never-smoking females. Further, they found that two PDCD1LG2 SNPs were associated with previous pulmonary tuberculosis infection. This is the first study to associate novel PD-L2 gene polymorphisms with lung cancer in females never-smokers and provides important insights into the pathways involved in lung cancer in females that do not smoke.

For lung cancer, the editors would recommend follow-up studies to further investigate sex differences with additional stratification factors such as a) occupational and environmental exposures; b) secondhand smoke risk of cancer in non-smokers who may be exposed to family members or co-workers who smoke; and c) stage of disease upon diagnosis. Such information will provide more comprehensive accounting of sex influences in cancer incidence as well as response to cancer treatment(s) and overall survival.

Using a large dataset consisting of 176,100 patients with glioma (124,502 glioblastoma and 51,598 lower grade gliomas) Stabellini et al. analyzed the differences in time to treatment between males and females. The authors found that male GBM patients had a statistically significant association only with >7 days to surgery (OR = 1.09, CI 1.05–1.13, *p* < 0.001) on the univariable model but this association was no longer present when using the multivariable model. Further, the study found that on average males receive more treatment than females, though this treatment may start later which may contribute to the differences observed in survival. The results were consistent with previous findings that GBM and LGG occurs more in males than in females and females tend to have better survival outcome. Further studies could be performed on additional datasets to validate these results and investigate additional factors associated with sex differences in time to treatment and clinical outcomes for GBM.

Overall, the themes in this Research Topic advocate that clinical, epidemiological and molecular differences exist by sex and are associated with sex-specific differences in clinical outcomes. Identifying sex-specific mechanisms that drive cancer could be leveraged for future therapies that move beyond using the same treatments for all cancer patients to tailoring treatments to the unique vulnerabilities of male versus female patients with cancer.

